# *FireLossRate*: An R package to estimate the loss rate of residential structures affected by wildfires at the Wildland Urban Interface

**DOI:** 10.1016/j.mex.2023.102238

**Published:** 2023-06-05

**Authors:** Vittorio Nicoletta, Raphaël D． Chavardès, Ahmad Abo El Ezz, Anne Cotton-Gagnon, Valérie Bélanger, Jonathan Boucher

**Affiliations:** a Laurentian Forestry Centre, Canadian Forest Service, Natural Resources Canada, Québec, QC, Canada; b Département de Gestion des Opérations et de la Logistique, HEC Montréal, Montréal, QC, Canada; c Atlantic Forestry Centre, Canadian Forest Service, Natural Resources Canada, Fredericton, NB, Canada; d École de Technologie Supérieure, Université du Québec, Montréal, QC, Canada

**Keywords:** Structural exposure, Wildland Urban Interface, WUI, Fire induced loss, Loss rate, Burn probability models, Burn-P3, Fire loss rate computation

## Abstract

To inform proactive management actions supporting community resilience to wildfires, we developed a new software package called *FireLossRate*. This package in R helps the user to compute wildfire impacts on residential structures at the Wildland Urban Interface (WUI). The package integrates spatial information about exposed structures, empirical equations that estimate the loss rate of structures affected by wildfires as a function of fireline intensity and distance from fire edge with fire growth modeling outputs from fire simulation software and burn probability models. *FireLossRate* helps to quantify and produce spatially explicit data on structural exposure and loss for single and multiple fires. The package automates post hoc analyses on simulations that include single or multiple wildfires and enables result mapping when combined with other packages available in R. In this paper, we describe the functionality of the *FireLossRate* package and introduce users to the interpretation of impact indicators of wildfires at the WUI. *FireLossRate* is available for download at https://github.com/LFCFireLab/FireLossRate.

• *FireLossRate* allows the computation of wildfire impacts indicators on residential structures at the Wildland Urban Interface in support of community fire risk management.

## Graphical abstract

**Figure:**
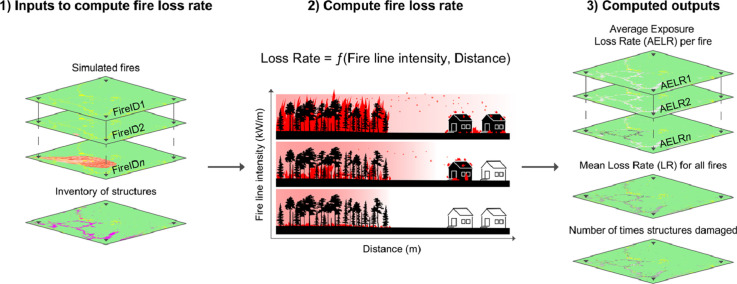

Specifications table

**Table:** 

Subject area:	Earth and Planetary Sciences
More specific subject area:	*Fire management; structural loss*
Name of your method:	*Fire loss rate computation*
Name and reference of original method:	*Abo El Ezz A, Boucher J, Cotton-Gagnon A, Godbout A (2022) Framework for spatial incident-level wildfire risk modeling to residential structures at the wildland urban interface. Fire Safety Journal 131 (103625), 1-14, doi:**10.1016/j.firesafe.2022.103625*.
Resource availability:	https://github.com/LFCFireLab/FireLossRate

## Method details

 

## Introduction

Forecasting wildfire impacts in communities is key to mitigate loss of life and infrastructure [1]. In many areas, the importance of such forecasts is heightened due to observed trends including longer and more catastrophic fire seasons [[2], [3]] and increasing human development in the Wildland Urban Interface (WUI) [4,5]. Combined, the changes to both the fire season and the WUI influence the exposure of residential structures (hereafter, “structures”) to wildfire impacts in many communities of the world [[6], [7]].

Addressing concerns about wildfire impacts on communities requires a rapidly usable and easy-to-disseminate tool like a software package. Ideally, the tool would help to estimate potential wildfire impacts to structures as a function of loss rate, i.e., susceptibility of structures as the percentage loss should they be exposed to a fire of a given intensity [[8], [9]]. Using spatially explicit information on structures along with fireline intensity outputs from fire growth or burn probability models like Burn-P3 [[1], [10]] can provide insights into structural exposure and loss for single and multiple fires across different spatial scales. Such insights can inform fire management and public safety agencies about single or multiple communities, or specific zones in communities that face higher potential exposure and loss due to fire convergence from higher ignition probabilities, hazardous fuel types, topographic effects, and prevailing wind directions. With these insights, proactive efforts to mitigate hazards and risks can be prioritized thereby enhancing community resilience to wildfires [11].

Insights on structural exposure and loss from fires across spatial scales are critical for fire managers; however, the ability to gain such insights remains rare given the paucity of available tools [8]. Although fire simulation software like Phoenix RapidFire [12] has been used for the estimation of structure loss in Australia, multiple inputs are needed to feed its underlying equations preventing its usage in conjunction with some fire behaviour models such as the Canadian Fire Behaviour Prediction System [13]. Some have developed software to assess wildfire risk to real estate parcels or to houses and infrastructure [14]; however, the software is proprietary and presents the same challenge in terms of fire behaviour modeling compatibility. To our knowledge there is no open-source software tool that can be used to forecast fire perimeters and fireline intensities from any fire behaviour model, and that integrates spatial information about residential structures to estimate wildfire related loss rates in different contexts, like operational decision support for single fire incident or multiple fire incident simulations within a wildfire risk assessment framework.

The objective of this work was to compute a suite of wildfire impact indicators on residential structures at the WUI using *FireLossRate*, a novel R package that applies a Structural Wildfire Risk Framework (SWRF) developed by Abo El Ezz et al. [8]. The SWRF supports the (i) computation of wildfire impacts on residential structures at the WUI using the Loss Rate functions of Abo El Ezz et al. [8], and (ii) their application in the context of deterministic single fire incidents or simulation-based burn probability models [1]. Usage and functionalities of the package were exemplified through two case studies, the first for a single fire incident, and the second for multiple fires generated using a burn probability model. We expect that the use of our package will support strategic planning management actions that increase community resilience to wildfires.

## Method description

### Structural Wildfire Risk Framework (SWRF)

The SWRF, as described by Abo El Ezz et al. [8], consists of four successive components that are represented by a hazard model, the structure inventory data (hereafter, “inventory data”), an exposure model, and an impact model. The hazard model spatially estimates burn probability and fireline intensity. In the Canadian context, the hazard model can be produced with fire simulation software like Prometheus [15] and Burn-P3 [10], both integrating the Fire Behaviour Prediction (FBP) System [13]. The inventory data along with the exposure model provide the location of structures and the degree to which these are directly or indirectly exposed to fire. Finally, the impact model applies response functions in terms of structure loss rate and outputs practical community scale impact indicators.

### Loss rate function

The Loss Rate function uses Fireline Intensity (*FLI*; measured in kW/m) and Distance of structures from the Fire Edge (*DFE*; measured in m) [8]. These empirical functions were developed using statistical analysis of loss rates estimated from a combination of sources, including fire expert opinion surveys and post-fire damage data from the international literature [[16], [17], [18], [19], [20]]. The function calculates the Loss Rate (*LR*;%) defined as the percentage of structures either lost or damaged from fires. For direct exposure, where structures are located within the WUI intermix zones, or zones where structures and wildland vegetation intermingle [21], the calculation is as follows:

(1) $$LR\left(\%\right)=4.94FLI^{0.34}$$

Where *LR* = 100 if *FLI* > 6800 kW/m. For indirect exposure, where structures are located within the WUI interface zones, or zones where areas with structures abut the wildland vegetation [21], *LR* is multiplied by a reducing factor based on distance from the fire edge defined as the Normalized Loss Rate (*NLR*):

(2) NLR=−0.21Ln(DFE)+1.37

Indirect exposure is applied only to structures that are located within a maximum distance of 500 m from the fire's edge.

### Impact indicators

The loss rate can be computed for each WUI pixel containing at least a structure, namely a Pixel with Structures (PWS). The following impacts indicators can then be produced. Below, 1–4 were proposed by Abo El Ezz et al. [8], and 5–6 were introduced in this work to support the probabilistic implementation of the SWRF:

1. Number of exposed structures, i.e., the count of PWS with a non-negative loss rate.
2. Number of lost structures, multiplying the structure count of each PWS by the associated loss rate.
3. Average exposure loss rate, dividing the number of lost structures by the number of exposed structures.
4. Average community loss rate, dividing the number of lost structures by the number of total structures.
5. Number of times a PWS was exposed within a defined maximum distance from the fire's edge.
6. Number of times a PWS was damaged by fire with a loss rate ≥50% following Federal Emergency Management Agency guidelines [22].

It is also possible to map a range of statistics representing the loss rate results, such as the mean, median, minimum, or maximum loss rate per exposed PWS.

### Implementation

Implementation of the *FireLossRate* package requires the following existing R packages: sf, raster, data.table, dplyr, and ggplot2. These packages support the reading and manipulation of a wide range of output file formats (including tabular files and spatial data like rasters and shapefiles) from fire growth modeling software like Prometheus [15] and Burn-P3 [10]. At its core, the implementation of the *FireLossRate* package consists of three main functions: *createStructureGrid, buildFires*, and *computeLR*. The three functions accept tabular files, rasters, and shapefiles. We hereafter describe these three main functions.

*A. createStructureGrid*

**Fig. 1:**
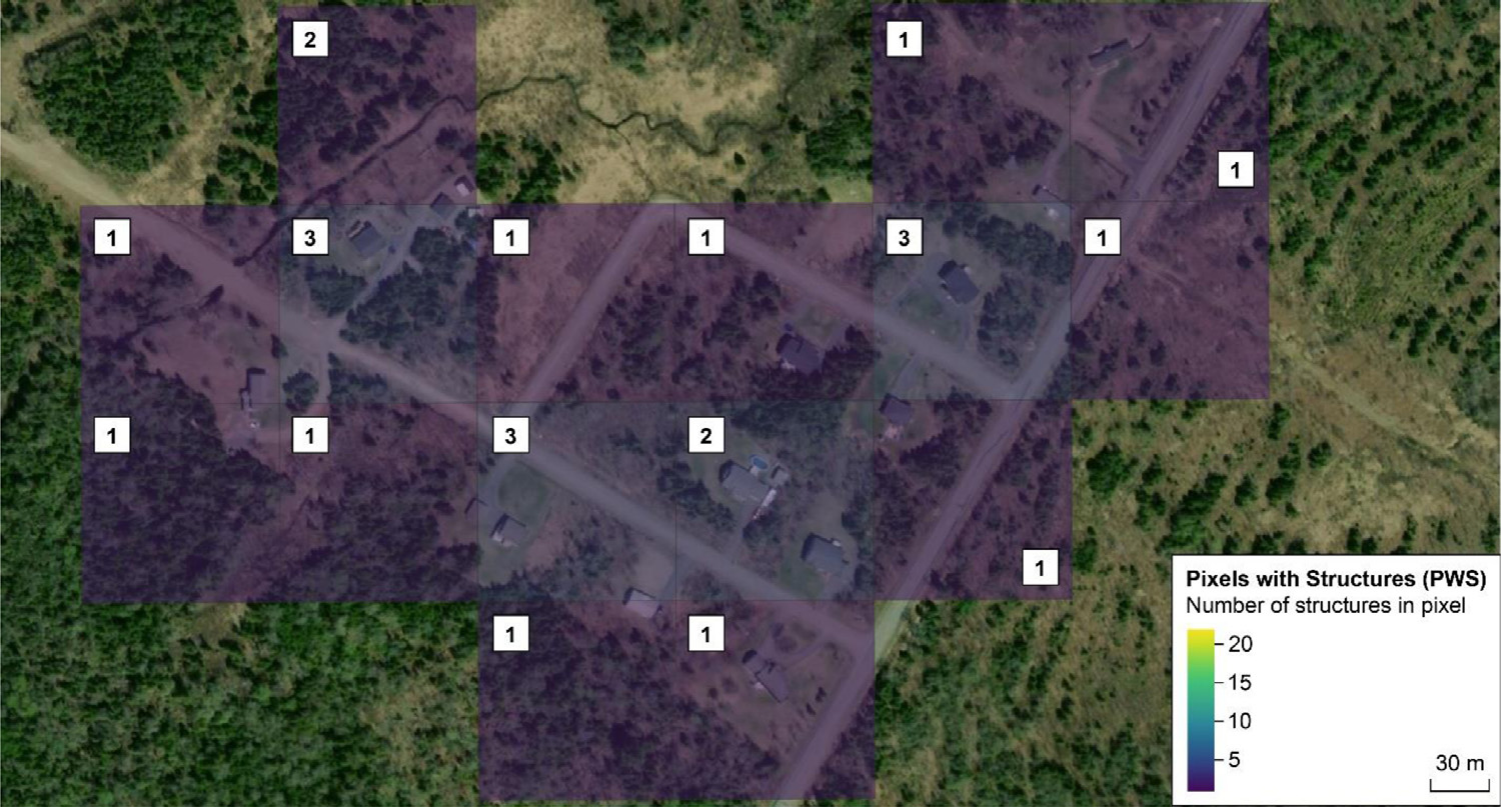
Example of *createStructureGrid* function's output using the *maximal* method: structures viewed from satellite imagery, the shapefile, and image of the grid. The count of structures for each pixel is shown in white boxes. Background source: Esri, i-cubed, USDA, USGS, AEX, GeoEye, Getmapping, Aerogrid, IGN, IGP, UPR-EGP, and the GIS User Community.

The *createStructureGrid* function prepares the inventory data about structures in the WUI ([8]; Fig. 1). Geospatial data of structures in the WUI need to be transposed onto a grid matching the fuel grid input into the fire growth modeling software. Given a shapefile containing the polygons of structures, a selected cell size, and a method to count structures, the *createStructureGrid* function produces a rectangular grid of Pixels with Structures (PWS) and the associated count. PWS are identified and associated with a structure count per PWS. Multiplying this count by the associated LR yields the expected number of burnt structures. Structures falling along the boundary of adjacent PWS warranted careful consideration. These structures could be counted multiple times whenever a portion of the structure intersected a PWS (*maximal* method), or only once within the PWS containing the largest area (*minimal* method). The *maximal* method produces the highest number of structures within the selected extent and may provide a more accurate estimate of exposure. In contrast, the *minimal* method produces the lowest and most accurate number of structures within the selected extent but potentially underestimates exposure. The user can focus on a specific extent, or study area, of that shapefile by providing four coordinates (xmin, ymin, xmax, ymax). The user can also decide to buffer and exclude from the count structures that are closer than a certain distance from the grid border. This exclusion addresses simulations that do not consider fires originating from outside of the selected area. For these simulations, structures near the grid border could be exposed to fewer fires than other structures, thus biasing the risk assessment.

Steps in the *createStructureGrid* function:

1. Create a bounding box of the shapefile or a portion of it.
2. Compute a grid of a given cell size from the bounding box.
3. Count the number of structures in each PWS.
  a. If the chosen method is *maximal*, count how many polygons of structures in the shapefile intersect each pixel.
  b. If the chosen method is *minimal*, identify in which pixel the largest surface of each polygon is contained, and count the structures in those pixels only.
4. If a certain distance from the border is specified, structures in the bordering area are excluded from the count.
5. Return the grid of PWS and the associated counts.

*B. buildFires*

The *buildFires* function supports the Hazard modeling component [8] with information about the position of the forecasted area burnt and intensity of single fire incidents or multiple fire incidents, such as those resulting from several iterations of a burn probability model. If the user already has a geospatial file with the position of the area burnt and fire intensity, then this function is not required. However, in the case of simulation outputs from Burn-P3, the user will have two large tabular files in wide format (meaning that information for a given pixel is stored in a single row of several columns) containing: (1) the fire intensity for each burnt pixel in each iteration; and (2) the iteration during which a given pixel burnt. To avoid multiple fires during the same iteration from overlapping, Burn-P3 should be parameterized to only simulate a single ignition per iteration. The *buildFires* function combines files (1) and (2) and produces a table with: Iteration, the position of burnt pixels given as Column and Row of the fire grid, and Fire Intensity (kW/m) in long format (i.e., information from a given pixel is stored in single columns of several rows). To account for file sizes and the large number of empty columns, the *buildFires* function modifies the *fread* function from the *data.table* package. The *buildFires* function takes as input the path to the file with Fire Intensity and the path to the files with the Iteration.

Steps in the *buildFires* function:

1. Read the Fire Intensity file.
2. Convert it from wide to long format.
3. Read the Iteration file.
4. Convert it from wide to long format.
5. Combine the two files.
6. Return a single file in long format with Iteration, Column, Row and Fire Intensity of burnt pixels.

*C. computeLR*

The *computeLR* function comprises the Exposure modeling, Impact modeling, and Risk simulation components of the Abo El Ezz et al. [8] framework for quantitative wildfire risk assessment to residential structures. The *computeLR* function is thus the engine of the *FireLossRate* package. From the information about the intensity and position of a given fire, and a grid of structures, *computeLR* identifies exposed structures and computes the associated loss rate. In addition to the two mandatory inputs, information about the given flame arrival time can be used to calculate more temporally explicit outcomes that are useful in the case of a single fire incident. Users can modify the maximum distance from fire edge to the centroid of PWS applied in the calculations, through the maxDist parameter (default is 500 m). The distance calculation was improved in *computeLR* with respect to the original calculation, as it now considers diagonal distances and uses distance from the edge of the fire pixel to the centre of the PWS, rather than edge to edge. The *computeLR* function also allows the user to change the parameters of the LR and NLR equations Eqs. (1) and (2), i.e., the factors, exponent, and constant.

Steps in the *computeLR* function:

1. Convert the fire intensity file to the appropriate format, if necessary.
2. Convert the flame arrival time file to the appropriate format, if necessary, then join with the fire intensity file.
3. Ensure same coordinates as the grid of structures.
4. Project fire position onto grid of structures.
5. Select PWS around a buffer twice the maximum distance to speed up computation.
6. Compute centroids of selected PWS.
7. Compute distance between PWS centroids and the Fire Edge.
8. For each PWS:
  a. Identify fire pixels closer than maxDist (there can be none).
  b. Compute LR and NLR.
  c. Select the pixel with maximum LR, considering both direct and indirect exposure.
9. Compute the number of total structures, namely the count of all PWS in the grid.
10. Compute the number of exposed structures, namely the count of PWS with a non-negative LR.
11. Compute the number of lost structures, multiplying the count of each PWS by the associated LR.
12. Compute the average exposure loss rate, dividing the number of lost structures by the number of exposed structures.
13. Compute the average community loss rate, dividing the number of lost structures by the number of total structures.
14. Return two files:
  a. Final: a list of the impacted PWS, namely those with a non-negative LR, and the associated fire intensity, position, and distance of the associated fire pixel.
  b. Out: a summary containing number of total structures, number of exposed structures, number of lost structures, average exposure loss rate, and average community loss rate.

## Applying *FireLossRate* to case studies

In this section, we show the application and different functions of the *FireLossRate* package through two case studies. The first case study considers a single fire incident, using a pre-computed structure grid. The second case study considers multiple fire iterations output from a Burn-P3 simulation, without a pre-computed structure grid.

### Single fire incident case study

We implemented the functions of the *FireLossRate* package for the single fire incident case study and compared our results to those presented by Abo El Ezz [8]. We also showed the usefulness of the package to assess impacts of a wildfire incident scenario on residential structures. We thus used the same case study scenario (scenario 1), at a resolution of 100 m, for a boreal community of Quebec, Canada.

In this case study, the hazard modeling component (i.e., fire extent and predicted intensity) of the SWRF was produced with the Prometheus software [15], a deterministic fire growth simulation model based on the FBP System [13]. A Prometheus scenario refers to the input data (fuel, topography, and weather data) and model parameters (e.g., simulation time frame, ignition coordinates, and date) for a single fire simulation that predicts fire growth. For the given scenario simulated in Prometheus, we obtained the necessary input files for *FireLossRate*, which consisted of two rasters, one indicating fire intensity for any burnt pixel (*fi.tif*), and one providing time at which the pixels burnt in the simulation (*flame-arrival-time.tif*). For the inventory data of the SWRF, we used Abo El Ezz et al.’s [8] 100 m resolution residential structure layer, with a count of structure per pixel, which was produced from the Microsoft Canadian Building Footprint database (MCBF 2020) coupled with visual validation using Google Maps and Esri Imagery. For more specific details about the scenario inputs and parameterization, please refer to Abo El Ezz et al. [8].

Three Prometheus output files were used as inputs to the *FireLossRate* analysis:

• *fi.tif*, a raster file with the Fire Intensity and position of the fire;
• *flame-arrival-time.tif*, a raster file with Flame Arrival Time for each position of the fire;
• *study-area.shp*, a shapefile containing a pre-computed structure grid and associated count for each PWS.

The code used to call the *computeLR* function is shown below (lines 1–7). A maximum distance of 500 m was passed to the function, while the default parameters for LR and NLR equations were adopted.

1 library(FireLossRate)

2 # Load data —-

3 fi <- raster('fi.tif')

4 flame_arrival_time <- raster('flame-arrival-time.tif')

5 structures <- read_sf('study-area.shp')

6 # Execution —-

7 final <- computeLR(fi, flame_arrival_time, structures, maxDist=500)

The *summaryLR* function is an auxiliary function that can be applied to a *computeLR* object to obtain a tabular output with results for each of the impact indicators proposed by Abo El Ezz et al. [8]: the number of exposed structures, number of lost structures, average exposure loss rate, and average community loss rate. Of the 400 structures in the simulated community, 128 were exposed to the fire, and 25 were lost. The average exposure loss rate and average community loss rate were 20% and 6%, respectively. Our values were slightly different from the results presented by Abo El Ezz et al. [8] (Table 1). The slight differences can be explained by a change in the distance calculation that was improved in *computeLR* as it now considers diagonal distances and uses distance from the edge of the fire pixel to the centre of the PWS, rather than edge to edge, as explained in the *Implementation* section.

**Table 1:** Comparison of impact indicators results obtained with computeLR and those obtained by Abo El Ezz et al. [8] for Scenario 1.

Impact indicators	*computeLR*	Abo El Ezz et al. [8]
Number of structures	400	400
Number of exposed structures	128	133
Number of lost structures	25	28
Average exposure loss rate (%)	20	21
Average community loss rate (%)	6	7

**Fig. 2:**
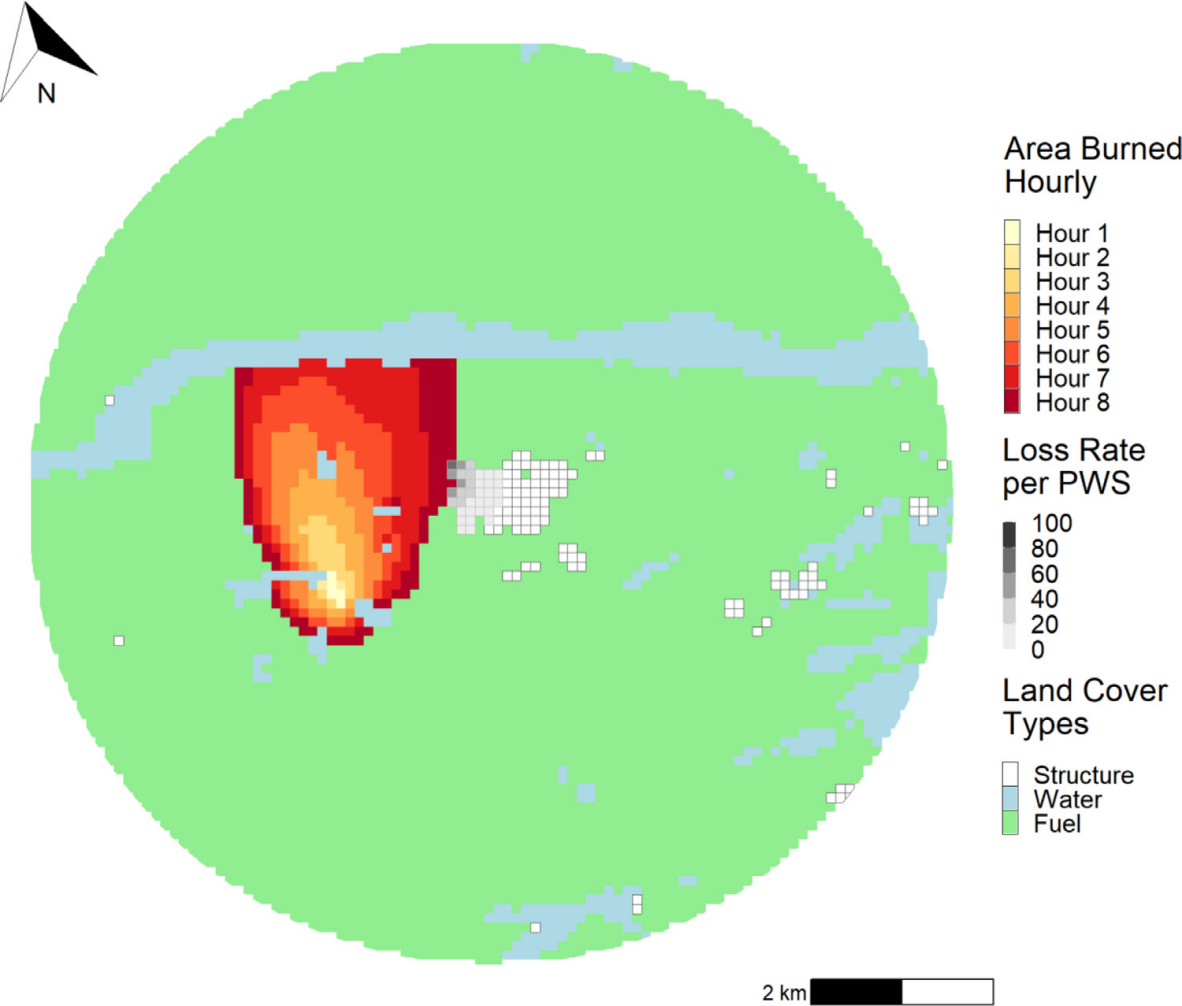
Study area considered for the application of the single fire and pre-computed structure grid using the *FireLossRate* package. A total of 400 structures represented the community. Results from the application yielded 128 exposed structures and 25 lost structures. The average exposure loss rate and average community loss rate were 20% and 6%, respectively. Land Cover Types shown were either water, fuel (forests and grasslands) or structures.

To visualize the results, we plotted the shapefile produced by the *computeLR* function. We added to this plot the hourly area burnt, and Land Cover Types (structure, water, or fuel) with the ggplot2 package. The resulting figure provides users with a spatial visualization of the average exposure loss rate per PWS (Fig. 2), which may be useful information for users who want to assess what portion of a community is more at risk of being affected by a wildfire.

### Multiple fires case study

The multiple fires case study aimed at highlighting the use of *FireLossRate* for a probabilistic risk assessment of residential structures in the WUI. This application of Abo El Ezz et al.’s [8] framework and loss functions were discussed in their paper, but not implemented. Here, we implemented the SWRF for probabilistic assessment, calculated the impact indicators, and suggested options on how to visualize them.

Before applying the *computeLR* function, we describe hereafter the selected study area, residential structure inventory data, the use of the *createStructureGrid* function, and the hazard modeling component of the SWRF accompanied with the *buildFires* function application. The analysis was conducted with the intention of assessing the risk posed by wildfire to residential structures for a specific region, considering the regional fire regime, climate, topography, and fuels. For this case study, Burn-P3 was selected as the fire hazard modeling engine [10],^1^, given its reliance on the FBP System [13].

#### Study area

The analysis was conducted within a 21.6 km × 24.3 km (52,488 ha) area in the province of New Brunswick, Canada. Regional climate is continental with maritime influences and labeled as Dfb (i.e., snowy winters, fully humid, and warm summers) in the Köppen-Geiger classification system [23]. Vegetation in the study area is predominantly closed mixedwood forests composed of red maple (*Acer rubrum* L.), sugar maple (*Acer saccharum* Marshall), yellow birch (*Betula alleghaniensis* Britt.), red spruce (*Picea rubens* Sarg.), balsam fir (*Abies balsamea* (L.) Mill.), eastern white pine (*Pinus strobus* L.), and eastern hemlock (*Tsuga canadensis* (L.) Carrière) [24]. The fire regime of this area is characterized by human-caused ignitions (79%), a low proportion of area burnt per year (burn rate) of 0.013%, and fires reaching a maximum size of 23,165 ha between 1980 and 2021 [25]. The area was a suitable candidate for applying the multiple fires function in *FireLossRate* because the landscape is dominated by wildland vegetation interspersed with small communities and countryside properties, thus representing adequately the intermix and interface configurations of the WUI [21]. Moreover, high quality geospatial inventory data of structures for this area was available.

#### Inventory data of structures

To identify the structures in the study area, we consulted the GeonNB data catalogue (http://www.snb.ca/geonb1/) and extracted the New Brunswick Buildings dataset (https://geonb.snb.ca/downloads/buildings/geonb_buildings-batiment_shp.zip). This dataset contains a collection of “*polygons representing a snapshot of buildings throughout New Brunswick. Data is derived from provincial LiDAR data acquired between 2015 and 2018 and include buildings present at the time of LiDAR collection*” [26]. The dataset is available under the Open Government License (http://www.snb.ca/e/2000/data-E.html). Data were last updated on 2021–10–19 and were downloaded as a shapefile. The dataset was then processed with the *createStructureGrid* function, as shown in the code below (lines 1–12).

1 library(FireLossRate)

2 library(sf)

3 library(raster)

4 library(dplyr)

5 # Create structure grid; the user needs to define the extent

6 structures <- read_sf('geonb_buildings-bâtiment_shp.shp')

7 structuresGrid <- createStructuresGrid(structures,

8                          cellSize = 100,

9                          mode = "maximal",

10                         xmin = userDefined, ymin = userDefined,

11                         xmax = userDefined, ymax = userDefined,

12                         internalBuffer = 500)

Steps were as follows. First, we called the required packages (lines 1–4), i.e., *FireLossRate, sf* for reading shapefiles, *raster* for reading rasters, and *dplyr* for the pipeline coding structures. We then imported the *geonb_buildings-bâtiment_shp.shp* file (line 6) and we created the structure grid (lines 7–12), specifying:

• a cellSize = 100 m;
• the *maximal* method, counting a structure each time it intersects a pixel;
• the coordinates of the area to consider;
• an internalBuffer = 500 m, excluding structures closer to 500 m from the border of the study area.

The *createStructureGrid* function led to the identification of a total structure count of 7923 in the study area minus the 500-m buffer.

#### Hazard modelling

Burn-P3 can include a Digital Elevation Model (DEM) to represent topography and estimate slope effects on simulated fire growth [10]. For the simulations, we used 100-m resolution elevation data from resampled aerial LiDAR derived rasters [26]. We also used a 100-m resolution layer of FBP System fuel types [13] to represent forest and grassland fuels, as well as water bodies and non-fuels in the study area (Supplementary Material Figs. S1 and S2).

In this scenario, the hazard modeling with Burn-P3 was parameterized to simulate burn perimeters and intensity of fires ≥1 ha, only during spring (i.e., leafless) conditions within the study area. Leafless conditions were selected because deciduous and mixedwood stands can support the highest rate of spread and fire intensity during spring [13], thus potentially placing structures more at risk. We used snowmelt timing maps from 1991 to 2020 produced with the ERA5 global reanalysis of the Copernicus Climate Change Service (C3S) [27] and phenology data derived from binary daily Normalized Difference Vegetation Index (NDVI) grids from 2002 to 2021 [28] to set spring leafless conditions during the fire season.

For our simulation, we used historical daily fire-weather data spanning from 1996 to 2021 that we obtained from four local meteorological stations to generate the fire-weather input to Burn-P3. This input included a list of daily noon records of temperature ( °C), relative humidity (%), wind direction (°) and speed (km/h), 24-h precipitation (mm), as well as indices of the Canadian Fire Weather Index System (FWI System), i.e., the Fine Fuel Moisture Code (FFMC), Duff Moisture Code (DMC), Drought Code (DC), Initial Spread Index (ISI), Buildup Index (BUI), and Fire Weather Index (FWI) [29].

The parameterization of the Burn-P3 simulation first consisted in applying spatially random ignitions in the study area. We used the sequential daily fire-weather selection method. The number of spread days (i.e., duration of burning) per fire was between one and three with the following probabilities: one day = 0.53, two days = 0.30, and three days = 0.17 adapted from Wang et al. [30] for the Canadian Eastern Temperate zone. The number of hours of burning per day was limited to 6 h (after [31]), and the percentage of matted grass curing was set to 85% (personal communication with the regional fire management authorities). We ran 5000 iterations with a single fire per iteration.

**Fig. 3:**
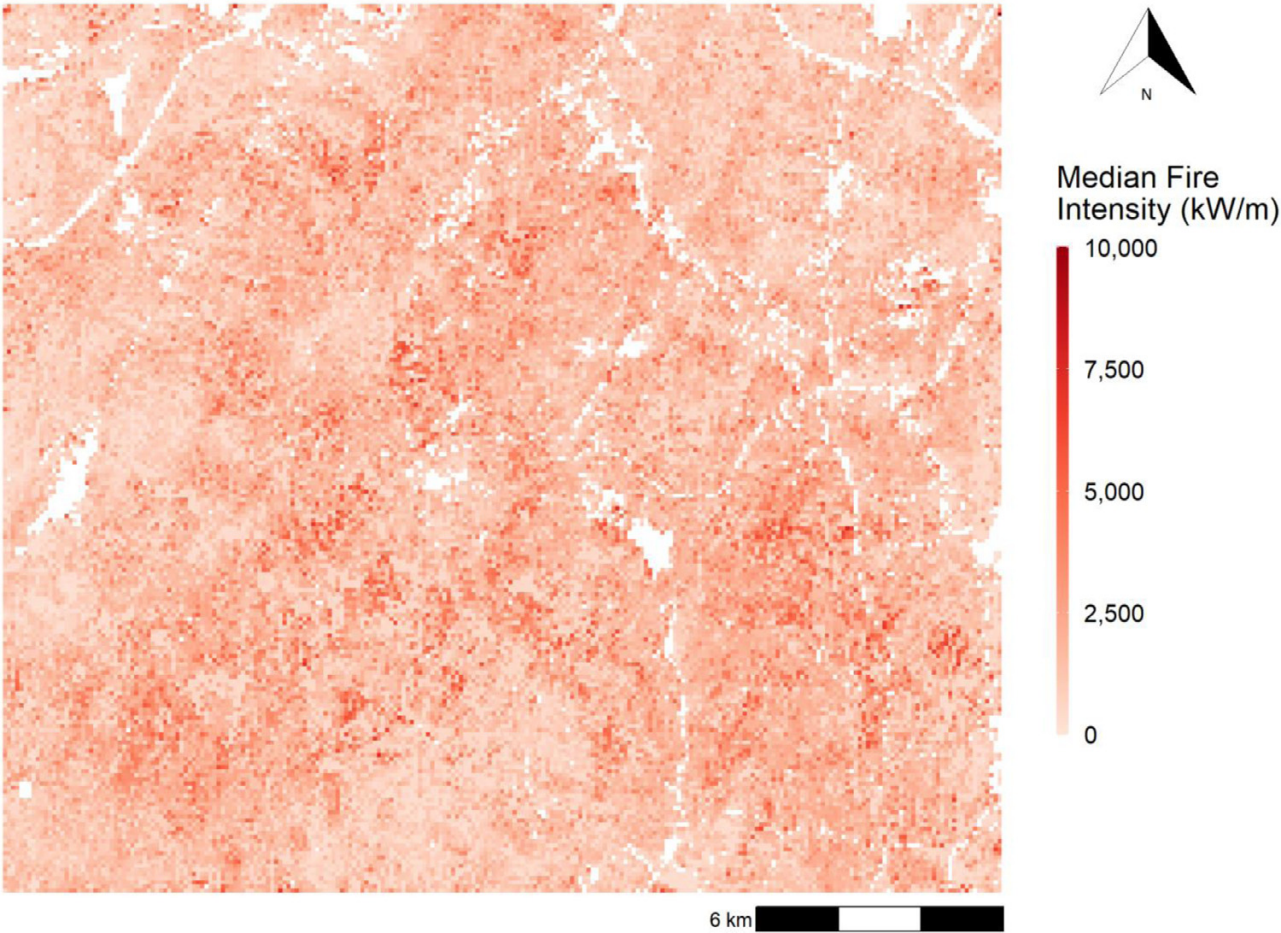
Median Fire Intensity across the 5000 iterations of the Burn-P3 simulation. Median Fire intensity describes the median rate of heat transfer of the fireline in kilowatt per metre (kW/m) for all simulated fires that burnt within each pixel.

The Burn-P3 simulation produced the following files, used as inputs for the *FireLossRate* package:

1. *BPmapFIRaw.csv*, containing Fire Intensity values, i.e., the rate of heat transfer of the fireline in kilowatt per metre (kW/m), for each burnt 100 m × 100 m pixel at a given iteration;
2. *BPmapBI.csv,* containing the iteration number in which each pixel burnt;
3. *BPmapFIMed.tif,* a raster file containing the median Fire Intensity value for each burnt pixel across all iterations, i.e., the median rate of heat transfer of the fireline in kilowatt per metre (kW/m) for all simulated fires that burnt within each pixel, along with information about the pixel size and the extent of the study area (Fig. 3).

The next step was to create the Fire List from the *BPmapFIRaw.csv* and *BPmapBI.csv* files using the *buildFires* function (lines 14–15). The output was a table with four columns (Table 2):

• FireID, identifying the iteration in which the fire was generated;
• column, specifying the column IDs in the grid where the fire spread;
• row, specifying the row IDs in the grid where the fire spread;
• fi, specifying the Fire intensity in the pixel identified by column and row for each fire.

**Table 2:** Example from the case study showing the first 10 rows of the fire list generated by the *buildFires* function.

FireID	column	row	fi (kW/m)
1	70	167	114
1	70	168	129
1	71	166	35
1	71	167	121
1	71	168	125
1	72	166	4
1	72	167	35
2	165	200	97
2	165	201	291
2	165	202	192

#### Exposure and impact modelling

To implement the exposure and impact modeling components of the SWRF through the *computeLR* function, we used files produced in the previous steps by the two other main functions (i.e., *createStructuresGrid* and *buildFires*) of the *FireLossRate* package. How to call the input files into *computeLR* and running the function itself are presented in the R code (lines 13–36). We then imported the *BPmapFIMed.tif* raster file (line 17) to ensure that the structure grid and the fire list had the same extent and the same coordinates as the Burn-P3 files (lines 18–27). Finally, we generated the *output* file by applying the *computeLR* function to each fire in the Fire List, regrouping them by FireID using the pipeline coding structure (lines 29–36).

13 # Create fire list

14 fireList <- buildFires('BPmapFIRaw.csv',

15                       'BPmapBI.csv')

16 # Convert fire list to spatial using Burn-P3 simulation info

17 fi <- raster('BPmapFIMed.tif')

18 structuresGrid <- st_transform(structuresGrid, st_crs(fi))

19 xmin <- fi@extent@xmin

20 ymin <- fi@extent@ymin

21 nCols <- fi@ncols

22 nRows <- fi@nrows

23 resolution <- (fi@extent@xmax - fi@extent@xmin) / fi@ncols

24 fireList$column <-xmin-resolution/2 + fireList$column * resolution

25 fireList$row <- ymin - resolution/2 + fireList$row * resolution

26 fireList <- st_as_sf(fireList, coords = c*(*2,3))

27 st_crs(fireList) <- st_crs(structuresGrid)

28 # Apply computeLR function to each fire using pipelines

29 output <- fireList%>%

30  group_by(fireID)%>%

31  group_map(

32    ∼ computeLR(

33      fi = .x,

34      buildings = structuresGrid,

35      maxDist = 500

36    ))

**Fig. 4:**
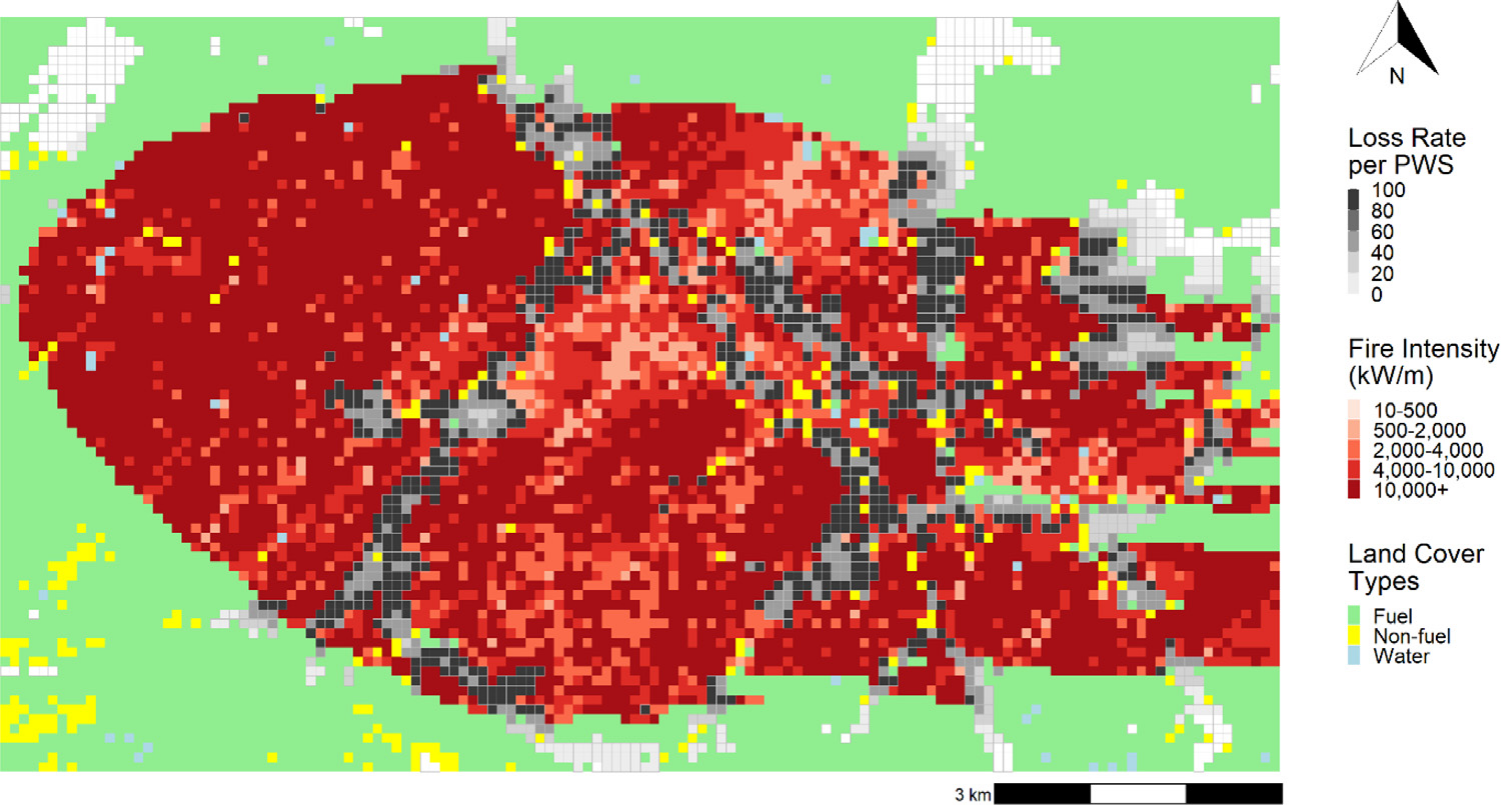
Loss Rate per Pixels with Structures (PWS) overlaid on the extent of the simulated fire and its fireline intensity (FireID 4850; red gradient). Unburned areas are shown as Land Cover Types with either fuel (forests and grasslands), non-fuel, or water.

The use of *computeLR* through the presented code produced two outputs: a summary table and a shapefile with the loss rate for each affected PWS per fire. The summary table contains five values for each fire: the total number of structures considered in the analysis (total structures), and the four impact indicators (i.e., number of exposed structures, number of lost structures, average exposure loss rate, and average community loss rate). This summary allows the examination of each simulated fire (5000 in our case) individually. For our current case study, the first five simulated fires did not impact any structures (Table 3). Simulated fires with FireID 7, 8, and 9 exposed less than ten structures each and none were lost. The fire with FireID 6 had 181 structures exposed with 5 lost, whereas 204 structures were exposed and 18 lost with the fire with FireID 10. This tabular format also allows data filtering to examine the most destructive simulated fire. In our case, it was FireID 4850, which burnt an area of 6040 ha, with 3708 exposed structures, 2163 lost structures, an average exposure loss rate of 58.3% and an average community loss rate of 27.3%. The shapefile output helps to examine how the impacts of FireID 4850 are spatially configured, as it provides the average exposure loss rate per PWS, as well as the Land Cover Type (structure, water, or fuel), and the fireline intensity for each burnt pixel (Fig. 4).

**Table 3:** Summary from the *computeLR* function for the first ten fires simulated in the multiple fires case study.

FireID	Total structures	Exposed structures	Lost structures	Average exposure loss rate	Average community loss rate
1	7923	0	0	0.00%	0.00%
2	7923	0	0	0.00%	0.00%
3	7923	0	0	0.00%	0.00%
4	7923	0	0	0.00%	0.00%
5	7923	0	0	0.00%	0.00%
6	7923	181	5	2.76%	0.06%
7	7923	8	0	0.00%	0.00%
8	7923	1	0	0.00%	0.00%
9	7923	9	0	0.00%	0.00%
10	7923	204	18	8.82%	0.23%

**Fig. 5:**
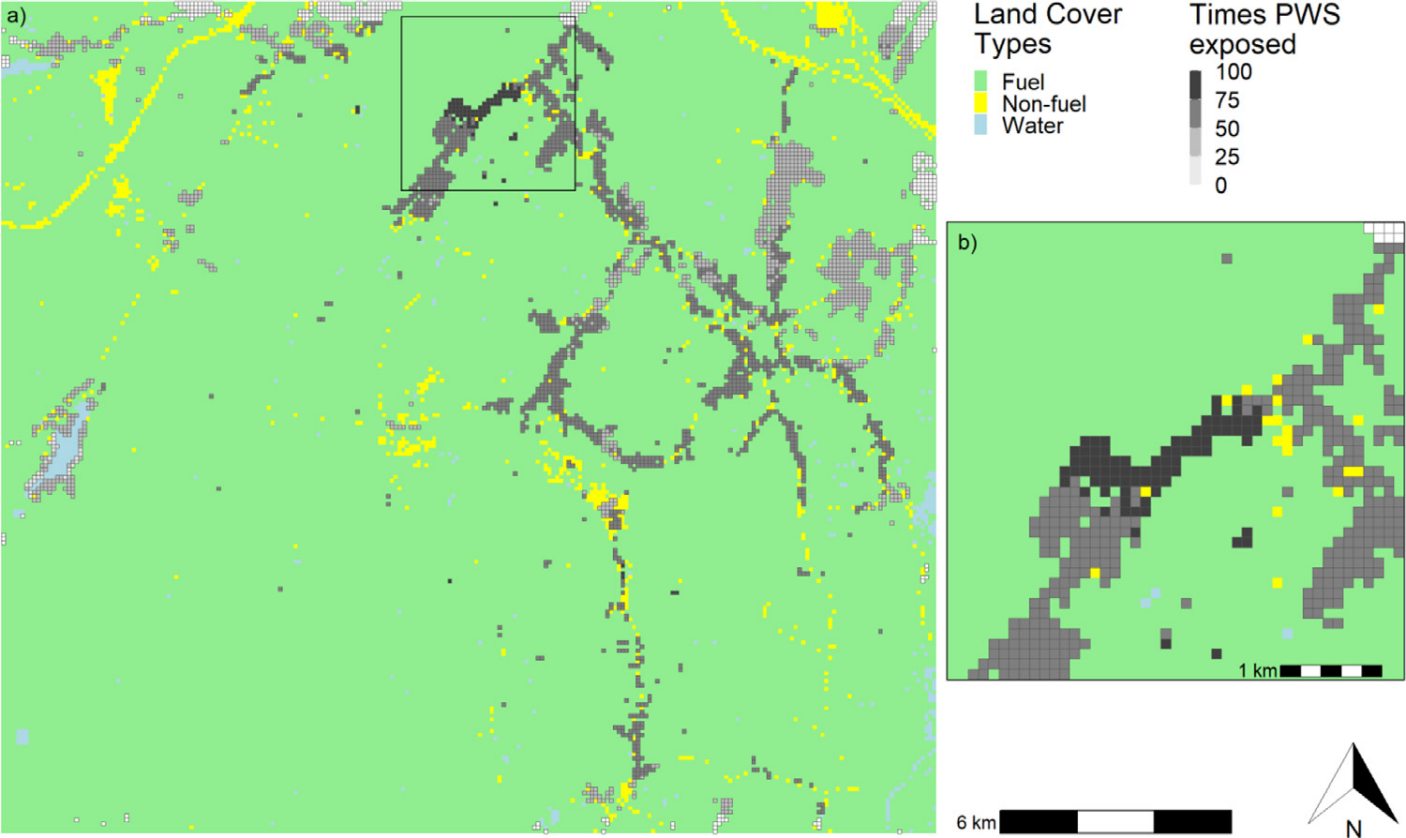
(a) Number of times Pixels with Structures (PWS) were exposed to fire (i.e., loss rate >0%), and (b) zoom of the area shown in the black rectangle. Land Cover Types were either fuel (forests and grasslands), non-fuel, or water.

**Fig. 6:**
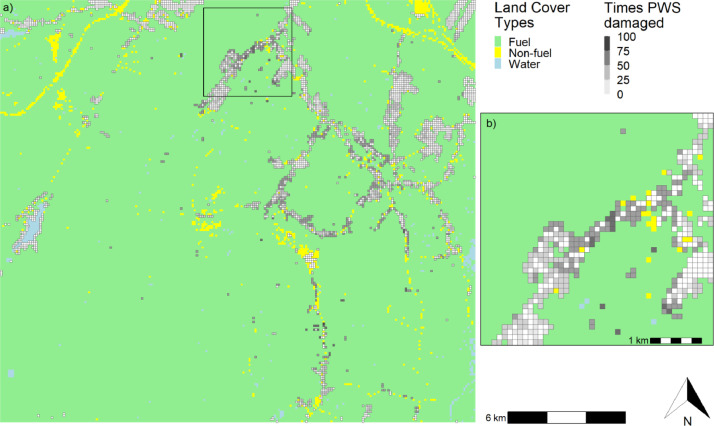
(a) Number of times Pixels with Structures (PWS) were damaged by fire, assuming structures were damaged if the associated loss rate was ≥50%, and (b) zoom of the area shown in the black rectangle. Land Cover Types were either fuel (forests and grasslands), non-fuel, or water.

To have a more comprehensive wildfire risk assessment for residential structures at community, landscape, or study area scale, we can create maps summarizing the number of times a PWS was exposed (i.e., within 500 m of a fire's edge) (Fig. 5) or damaged by fire (i.e., with a loss rate ≥50% following Federal Emergency Management Agency guidelines [22], or a specific threshold determined by the user) (Fig. 6). It is also possible to map a range of statistics representing the loss rate results, such as the mean, median, minimum, or maximum loss rate per exposed PWS during the simulation, and the number of structures damaged within each PWS across iterations (Supplementary Materials S3–S7).

## Discussion

*FireLossRate* is a novel package that operationalizes the conceptual framework and empirical loss functions proposed in Abo El Ezz et al. [8]. Through the single fire incident case study, we demonstrated how the *FireLossRate* package enables the calculation of potential impacts to structures, as a function of fire behaviour, namely FLI and DFE, as well as structural exposure and loss rates. The package also enables the use of probabilistic fire growth modeling as exemplified by the multiple fire incidents case study. The latter offered a comprehensive assessment of fire risk to structures through the inclusion of fire occurrence and likelihood derived from a simulation-based burn probability model. This capacity of the *FireLossRate* package to handle multiple fire incidents allows the synthesis of a suite of impact indicators in tabular and graphical formats that can be useful for a range of stakeholders including public safety and land managers. For example, mapping areas with potentially higher rates of structure loss offers insights to a range of concerned parties, from governments to homeowners, on where to prioritize structural retrofitting or mitigation efforts (e.g., FireSmart; [32]). Such information can also support the planning and design of new neighbourhoods and housing developments that are less vulnerable to fires.

The *FireLossRate* package facilitates the application of the SWRF by automated quantification of potential impacts on residential structures using a suite of impact indicators. The package is an accessible, open-source, and flexible tool provided geospatial inventory data of structures and fire hazard modeling (i.e., spatialized FLI). We ensured that the package maintains spatial resolution adaptability. Moreover, *FireLossRate* is adaptable to any fire growth modeling tool that provides spatialized FLI in kW/m. We improved the distance calculation and added two options for structure count in PWS. A current limitation of the package is that there is only one loss rate function applied. However, as additional loss functions are validated and improved over time, we aim to implement these in the package as they become available. Another limitation is that the default maxDist of 500 m restrains the package to model damages to any further PWS, while there have been observations of damage at larger distances [33]. In the meantime, the package allows for user defined parameterization for the LR and NLR equations Eqs. (1) and (2) and distance from fire's edge. Other limitations include the selection of the spatial resolution, which could affect the fuel typing, area burnt, and hence the fireline intensity output from fire growth simulations. In addition, PWS are only characterized by a single loss rate value and do not account for variability in the degree of fire vulnerability of residential structures according to different construction systems and vegetation conditions within the structure ignition zone.

## Conclusion

We presented and discussed the methodology and computational procedures behind *FireLossRate*, a newly developed open-source package, which integrates structure inventory data and empirical loss rate functions with results from fire growth modeling software to estimate a suite of wildfire impact indicators on residential structures. The applicability of *FireLossRate* was demonstrated with two case studies in Canada including an incident level single scenario event and a probabilistic fire impact assessment. The complementarity of the *FireLossRate* package with outputs from fire growth modeling software like Prometheus and Burn-P3 means that wildfire impacts on residential structures can be estimated for historical and current periods, and predicted for future periods provided the availability of future fire-weather data and fuel layers [31]. In both case studies, historical fire-weather data was used to predict fire perimeters and fireline intensities. However, a user can generate daily weather for future periods using normals derived from Global Climate Models thereby providing insights on wildfire impacts to residential structures due to climate change. Given the flexibility of the package, we encourage users to explore other approaches to output their results according to their specific applications that may not be limited to residential structures, or alternatively users can focus on outputs of the *createStructureGrid* function. For example, users could apply this package to investigate socioeconomic impacts associated with fire events such as economic losses due to reconstruction, short-term and long-term population displacement, and shelter needs.

## Ethics statements

No ethics statement necessary.

## CRediT authorship contribution statement

**Vittorio Nicoletta:** Conceptualization, Methodology, Software, Validation, Formal analysis, Investigation, Data curation, Writing – original draft, Writing – review & editing, Visualization. **Raphaël D． Chavardès:** Formal analysis, Investigation, Data curation, Writing – original draft, Writing – review & editing, Visualization. **Ahmad Abo El Ezz:** Conceptualization, Methodology, Writing – review & editing, Visualization. **Anne Cotton-Gagnon:** Conceptualization, Writing – review & editing, Visualization. **Valérie Bélanger:** Writing – review & editing, Supervision. **Jonathan Boucher:** Conceptualization, Methodology, Writing – original draft, Writing – review & editing, Visualization, Supervision, Project administration.

## Declaration of Competing Interest

The authors declare that they have no known competing financial interests or personal relationships that could have appeared to influence the work reported in this paper.

## Data Availability

Data will be made available on request.

## Appendix Supplementary materials

Image, application 1
