# Supplementary material for: FireLossRate: An R package to estimate the loss rate of residential structures affected by wildfires at the Wildland Urban Interface
Source: MethodsX. 2023 Jun 5;10:102238. doi: 10.1016/j.mex.2023.102238 (PMC10326497; doi:10.1016/j.mex.2023.102238)
Supplement: Supplementary file 1 [file mmc1.docx]

**Supplementary Material**


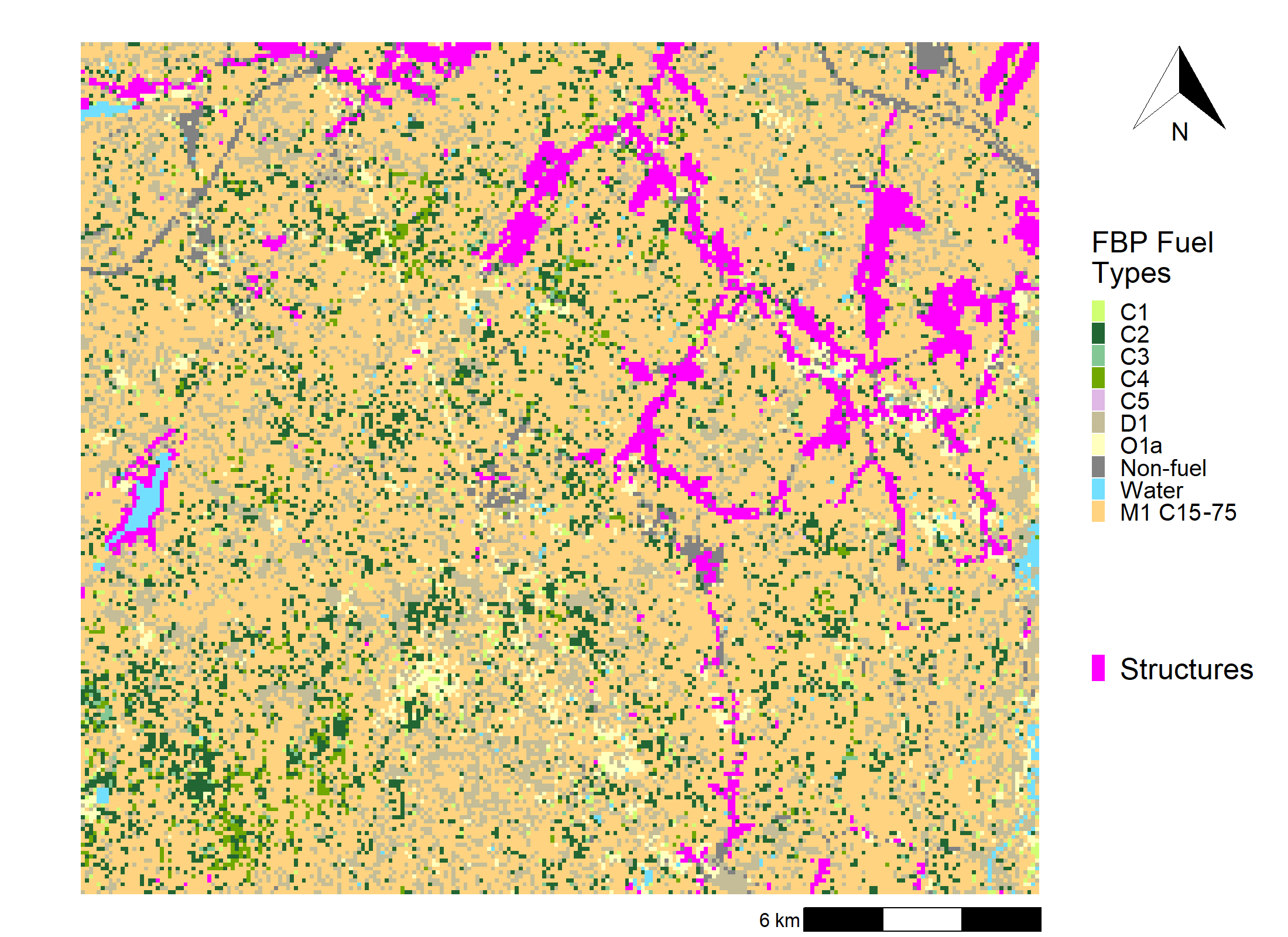


**Figure S1.** Fire Behaviour Prediction (FBP) Fuel Types and residential structures. C2 = Boreal Spruce, C3 = Mature Jack and Lodgepole Pine, C4 = Immature Jack and Lodgepole Pine, C5 = Red and White Pine, C6 = Conifer Plantation, D1 = Leafless Aspen, O1a = Matted Grass, M1 C15-75 = Boreal Mixedwood – Leafless (15-75% Conifer) (Forestry Canada Fire Danger Group 1992).


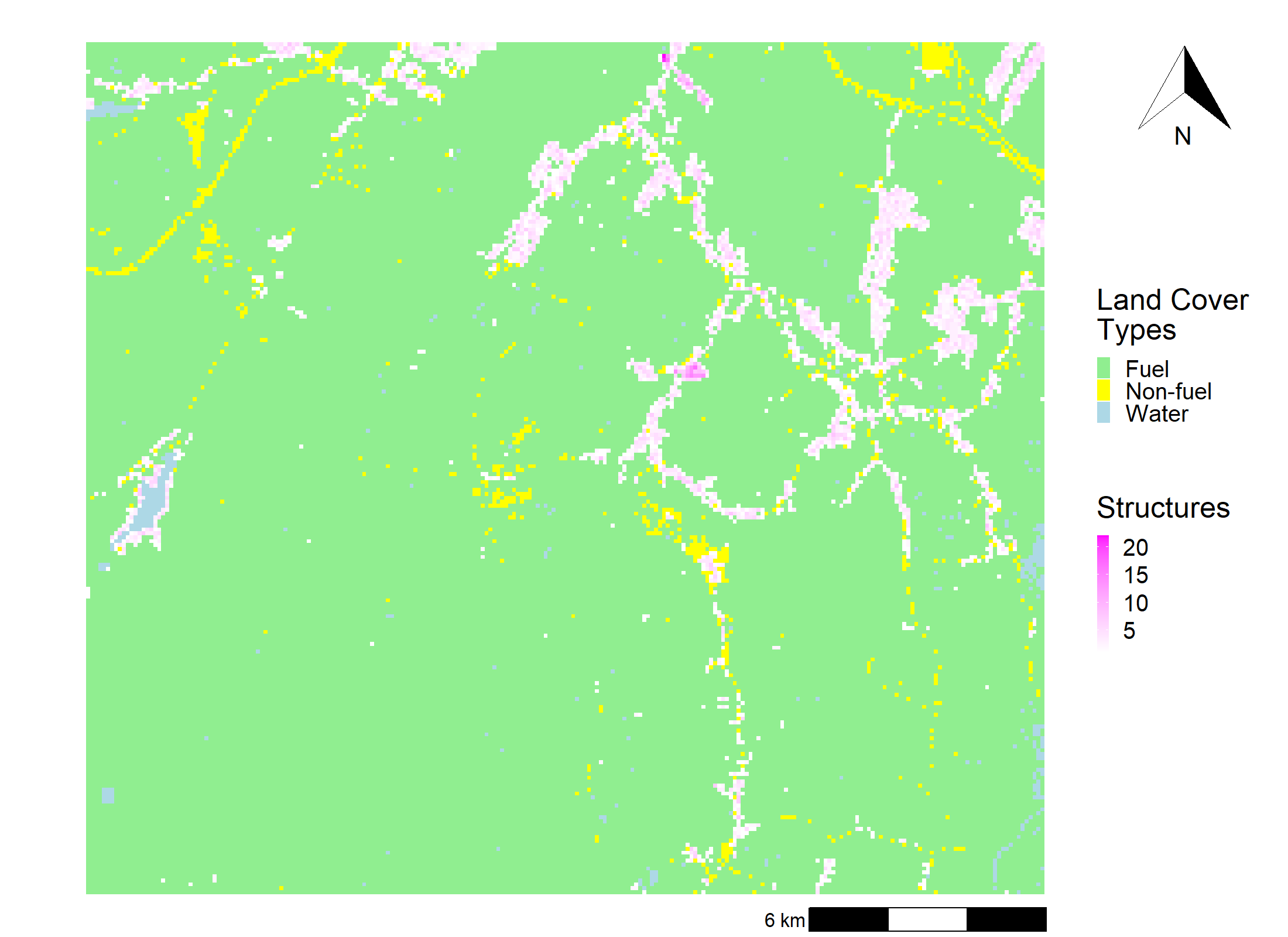


**Figure S2.** Land Cover Types and number of structures per Pixels with Structures. Land Cover Types were either fuel (forests and grasslands), non-fuel, or water.


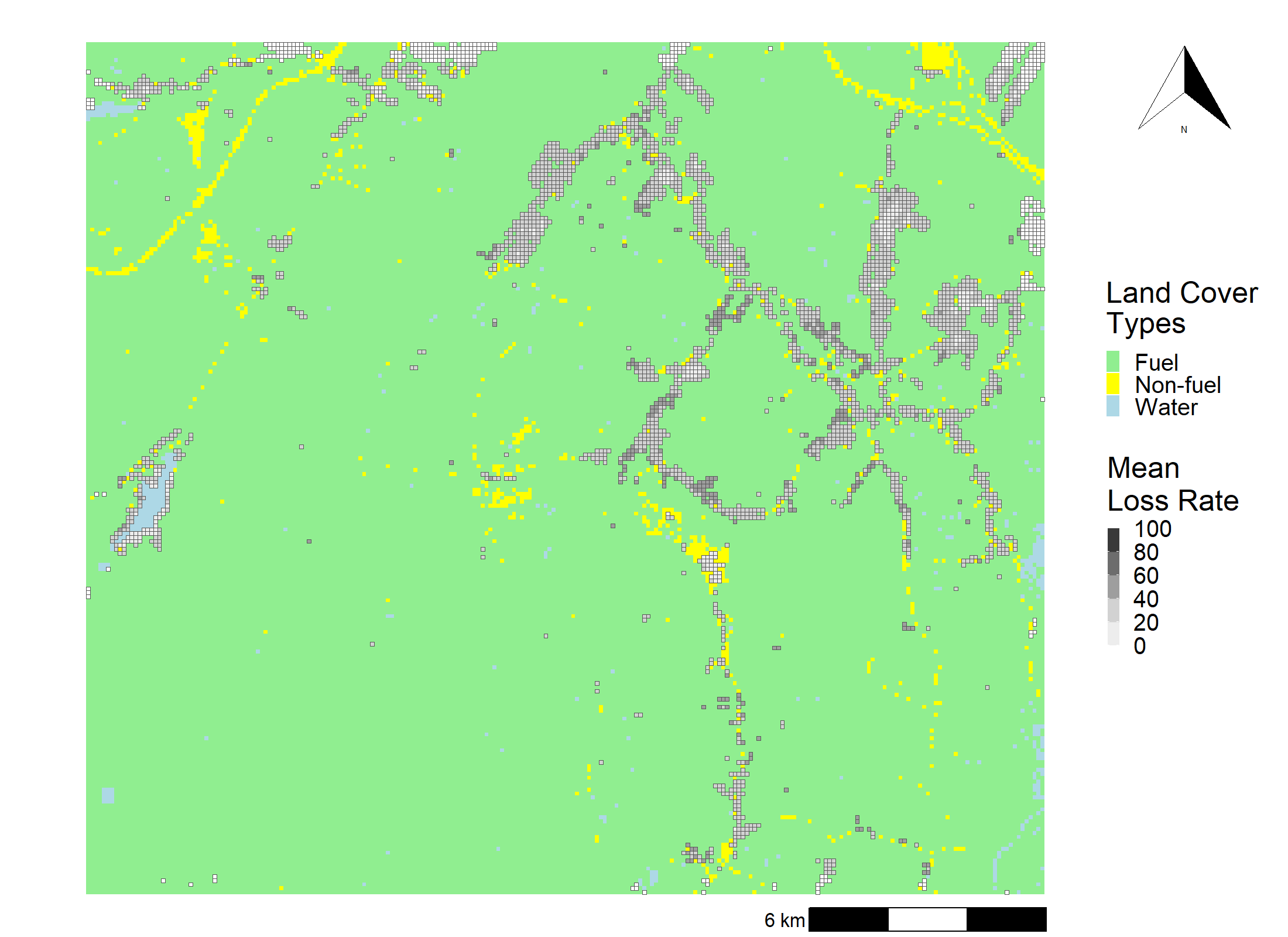


**Figure S3**. Mean Loss Rate per Pixels with Structures (PWS) exposed to fire. Land Cover Types were either fuel (forests and grasslands), non-fuel, or water.


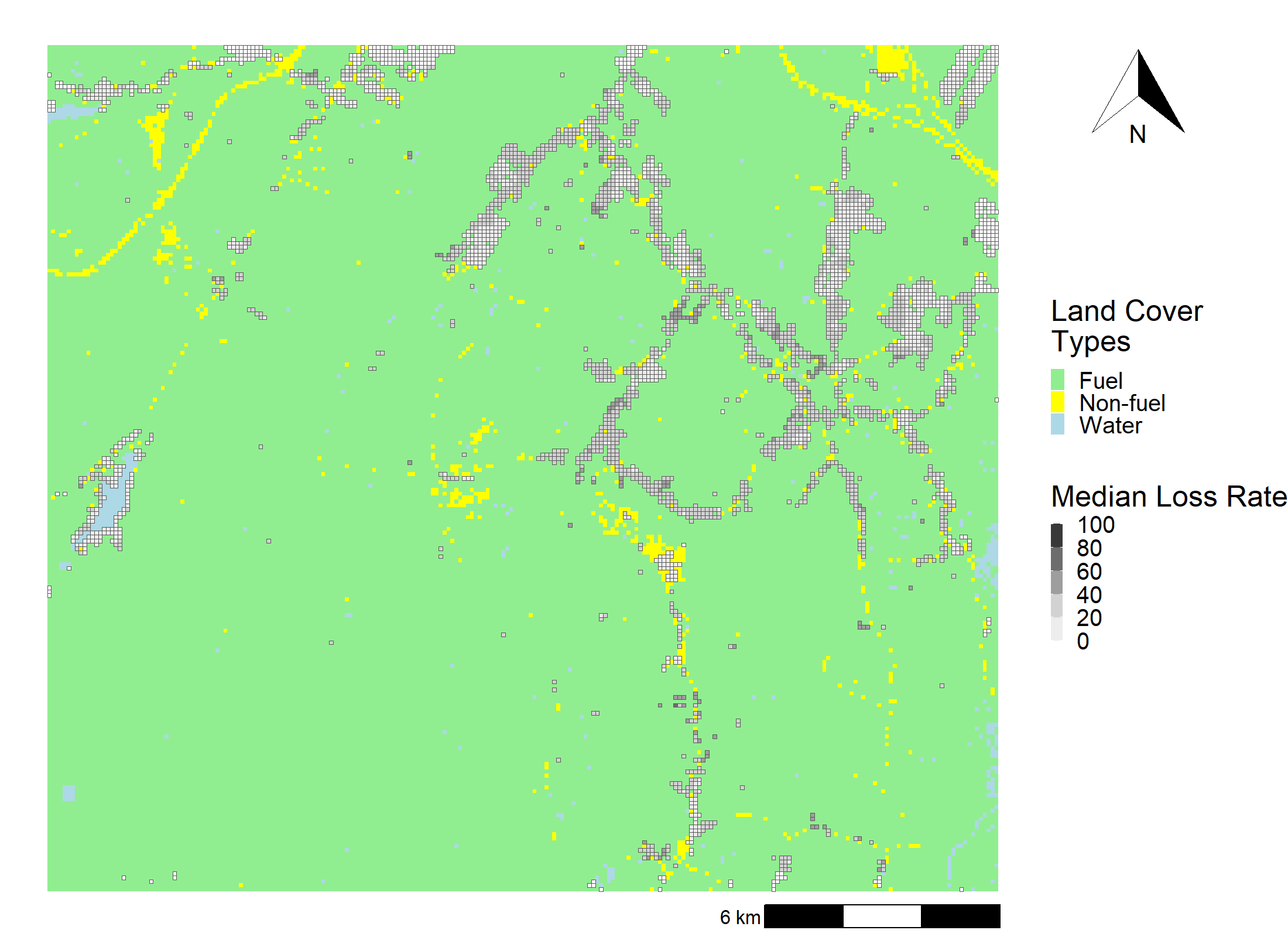


**Figure S4**. Median Loss Rate per Pixels with Structures (PWS) exposed to fire. Land Cover Types were either fuel (forests and grasslands), non-fuel, or water.


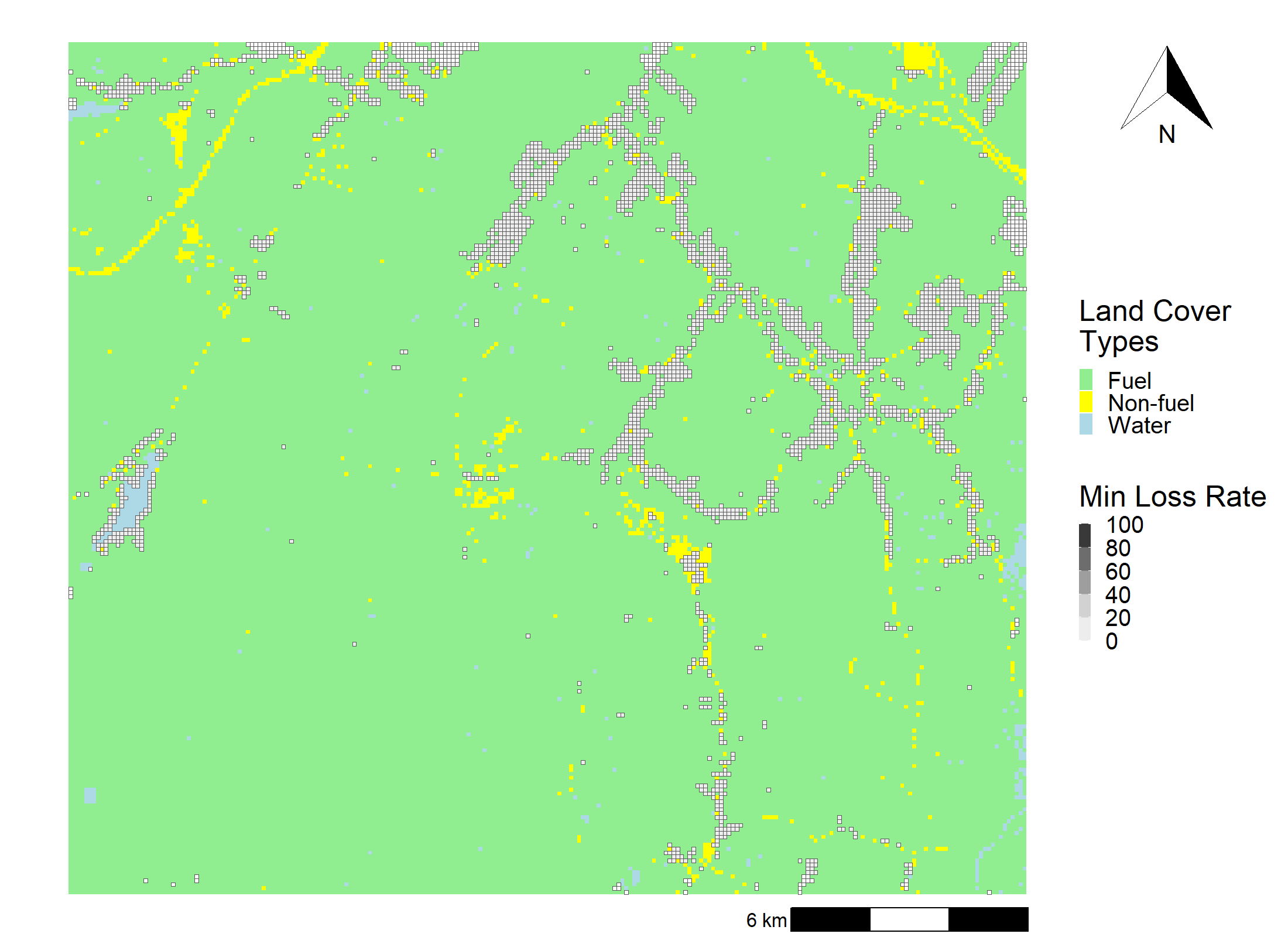


**Figure S5**. Minimum Loss Rate per Pixels with Structures (PWS) exposed to fire. Land Cover Types were either fuel (forests and grasslands), non-fuel, or water.


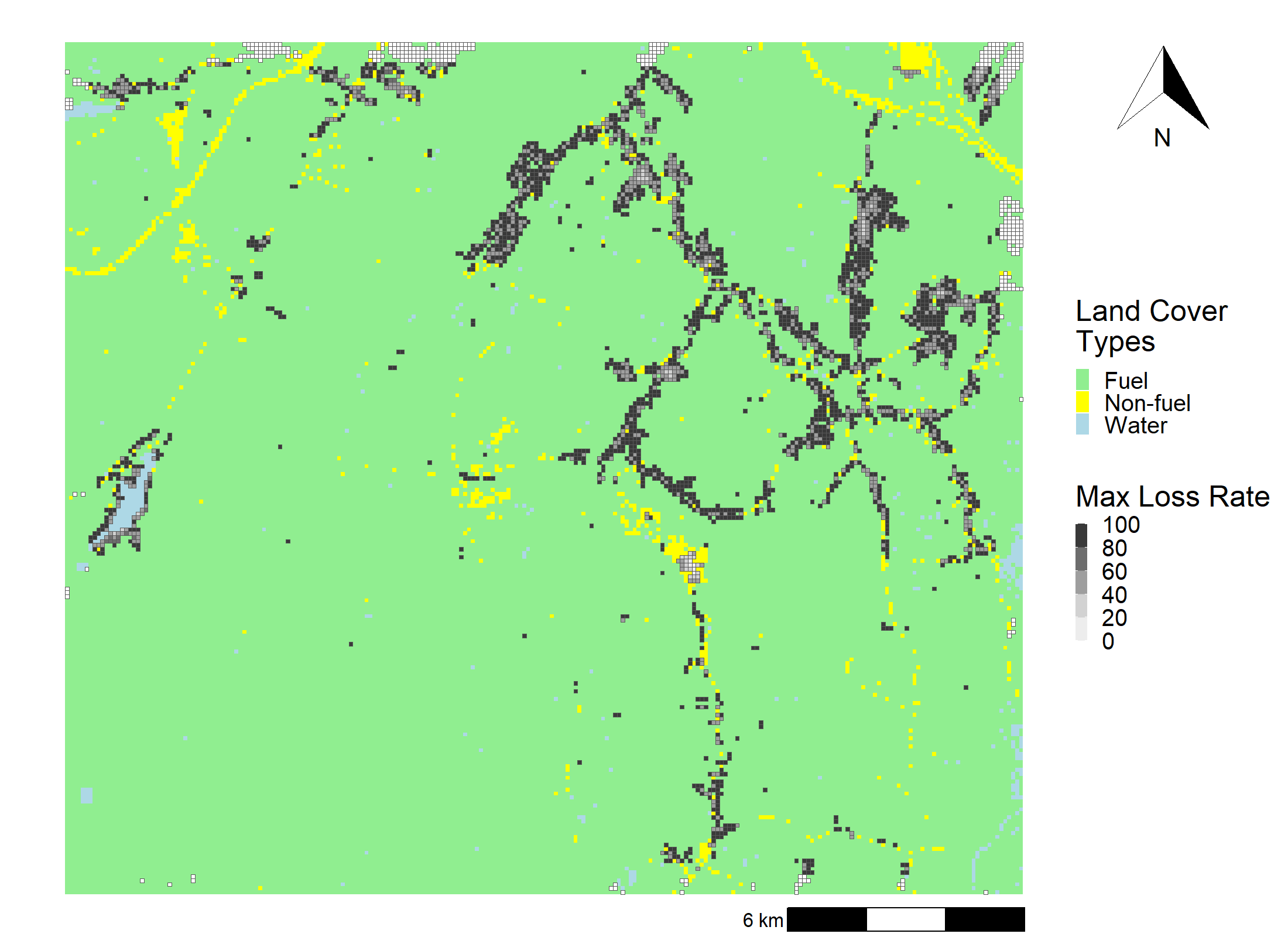


**Figure S6.** Maximum Loss Rate per Pixels with Structures (PWS) exposed to fire. Land Cover Types were either fuel (forests and grasslands), non-fuel, or water.


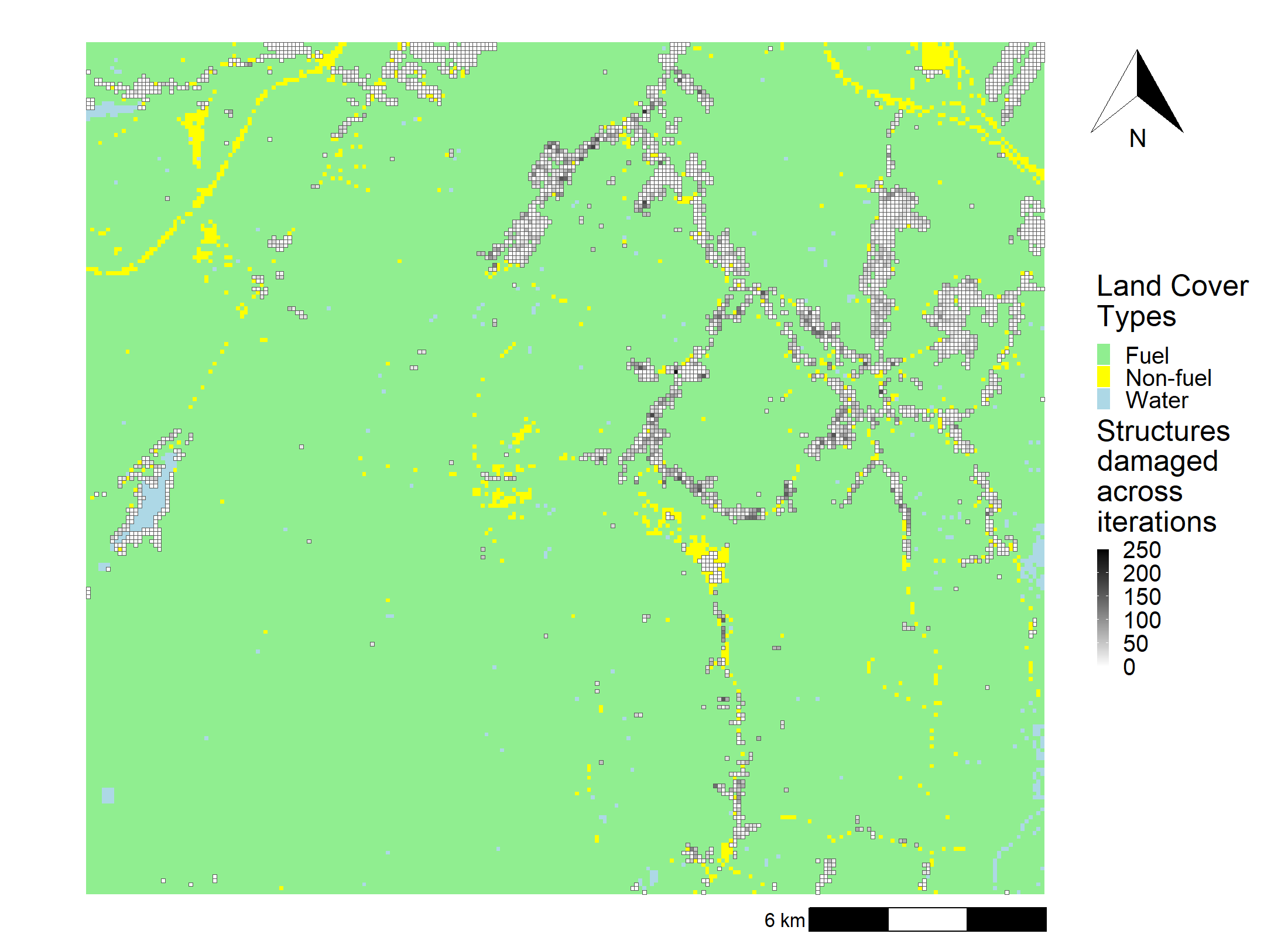


**Figure S7.** Number of structures damaged within each Pixel with Structures (PWS) across iterations computed as the number of structures within each PWS multiplied by the number of times the PWS was damaged, assuming structures were damaged if the associated loss rate was ≥50%. Land Cover Types were either fuel (forests and grasslands), non-fuel, or water.
